# Clinical Application of a Customized Gene Panel for Identifying Autism Spectrum Disorder-Associated Variants

**DOI:** 10.3390/medicina61071273

**Published:** 2025-07-14

**Authors:** Vittoria Greco, Donatella Greco, Simone Treccarichi, Maria Bottitta, Pinella Failla, Antonino Musumeci, Carla Papa, Valeria Chiavetta, Francesco Calì, Mirella Vinci

**Affiliations:** Oasi Research Institute-IRCCS, 94018 Troina, Italy; vgreco@oasi.en.it (V.G.); dgreco@oasi.en.it (D.G.); streccarichi@oasi.en.it (S.T.); mabot@oasi.en.it (M.B.); pfailla@oasi.en.it (P.F.); amusumeci@oasi.en.it (A.M.); cpapa@oasi.en.it (C.P.); vchiavetta@oasi.en.it (V.C.); mvinci@oasi.en.it (M.V.)

**Keywords:** target genetic panel, next-generation sequencing, SFARI database, novel variant identification

## Abstract

*Background and Objectives*: Autism spectrum disorder (ASD) is a neurodevelopmental disorder that belong to genetic and epigenetic mechanism. Despite the recent advantages in next-generation sequencing (NGS) technology, ASD etiology is still unclear. *Materials and Methods*: In this study, we tested a customized target genetic panel consisting of 74 genes in a cohort of 53 ASD individuals. The tested panel was designed from the SFARI database. *Results*: Among 53 patients analyzed using a targeted genetic panel, 102 rare variants were identified, with nine individuals carrying likely pathogenic or pathogenic variants considered genetically “positive.” We identified six de novo variants across five genes (*POGZ* 2 variants, *NCOR1*, *CHD2*, *ADNP*, and *GRIN2B*), including two variants of uncertain significance in *POGZ* p.Thr451Met and *NCOR1* p.Glu1137Lys, one likely pathogenic variant in *GRIN2B* p.Leu714Gln, and three pathogenic variants in *POGZ* p.Leu775Valfs32, *CHD2* p.Thr1108Metfs8, and *ADNP* p.Pro5Argfs*2. *Conclusions*: This study presents a comprehensive characterization of the targeted gene panel used for genetic analysis, while critically evaluating its diagnostic limitations within the context of contemporary genomic approaches. A pivotal accomplishment of this study was the ClinVar submission of novel de novo variants which expands the documented mutational spectrum of ASD-associated genes and enhances future diagnostic interpretation.

## 1. Introduction

Autism spectrum disorder (ASD), as defined by the DSM-5, is a clinically and etiologically heterogeneous neurodevelopmental condition characterized by variable symptom severity and diverse genetic underpinnings [1,2]. According to the DSM-5, ASD is diagnosed based on challenges in social communication and the presence of restricted or repetitive behaviors. The condition is classified into three levels of severity, reflecting the degree of support required. Level 1 represents mild ASD, where individuals may have social difficulties and prefer routines but require minimal support. Level 2 involves more pronounced challenges in communication and behavior, often requiring substantial support. Level 3 is the most severe, with significant impairments in verbal and nonverbal communication, rigid behaviors, and high dependency on very substantial support for daily functioning [3].

Individuals presenting with ASD tend to utilize healthcare services more frequently than those without disabilities, resulting in substantial medical costs for both families and healthcare systems [4]. These challenges highlight the need for policy interventions aimed at reducing the financial burden on families of individuals with ASD, especially in countries without tax-based or universal health coverage systems.

Growing evidence underscores the substantial genetic contribution to ASD pathogenesis, with recent advances in molecular diagnostics enabling more precise identification of causal variants. Modern genomic approaches—including chromosomal microarray (array-CGH), targeted gene panels, and whole exome sequencing (WES)—have become indispensable tools for ASD evaluation, each offering distinct advantages for detecting pathogenic mutations across the spectrum of disease-associated loci [5,6]. These technological developments have significantly enhanced our ability to unravel ASD’s complex genetic architecture while improving diagnostic yield in clinical practice. Despite significant advancements in next-generation sequencing (NGS) technologies, the diagnostic yield for detecting genetic variants causative of ASD remains challenging, typically below 30% in clinical settings [7,8,9]. This limitation persists due to several factors: (1) the extreme genetic heterogeneity of ASD, involving hundreds of risk genes with variable penetrance; (2) the substantial contribution of non-coding variants and structural variations not routinely captured by standard sequencing approaches; and (3) the complex interplay between genetic predisposition and environmental influences. While WES detects pathogenic variants in ∼30% of ASD cases, the remaining molecularly undefined patients necessitate WGS, functional studies, and combined diagnostic genetic tests [10,11].

Given the wide genetic heterogeneity of ASD, the Simons Foundation Autism Research Initiative (SFARI) gene database serves as a valuable resource to help elucidate the molecular etiology of the disorder. One increasingly popular approach involves integrating gene expression data with clinical genetic findings. This database comprises lists of curated genes (SFARI-genes) considered to have causative roles in ASD when mutated in patients [12,13,14]. According to the Q1 2025 Release Notes, the SFARI Gene database includes 1136 scored genes and 94 uncategorized ones. Based on the statistical association between genes and ASD, SFARI employs a scoring system: score 1 is assigned to genes with high-confidence evidence of ASD involvement, score 2 to strong candidate genes, and score 3 to genes with relatively weaker supporting evidence. Additionally, genes with well-established links to syndromic forms of ASD are categorized as score S (syndromic).

This study characterizes the diagnostic performance of a targeted next-generation sequencing (NGS) panel, designed based on ASD-associated genes from the SFARI database, in a cohort of 53 unrelated individuals with autism spectrum disorder. Through comprehensive variant analysis, we evaluate the panel’s diagnostic yield and clinical utility, while highlighting the critical role of NGS technologies in modern ASD genetic diagnostics.

## 2. Materials and Methods

### 2.1. Patients Recruitment and Inclusion Criteria

A cohort of 53 unrelated individuals, with a mean age of 12.5 (±4.5) years, all diagnosed with ASD, was examined in this study. A total of 45 males and 8 females underwent the genetic test. The consecutive patients were recruited starting in 2018 from the Oasi Research Institute–IRCCS of Troina (Sicily, Italy, EN). Prior to genetic testing, all participants underwent genetic counselling. All the subjects, or their legal guardians, gave written informed consent before their inclusion in the experimental protocol. The study was conducted in accordance with the Declaration of Helsinki of 1964 and its later amendments and was approved by the Ethics Committee of the Oasi Research Institute–IRCCS of Troina, Italy (approval code: 2018/07/18/CE-IRCCS-OASI/14). Inclusion criteria for patient selection required a confirmed ASD diagnosis based on the DSM-5 diagnostic criteria, encompassing all three levels of severity. Inclusion criteria required a diagnosis of ASD according to DSM-5, including (A) persistent deficits in social communication and interaction across multiple contexts; (B) restricted, repetitive patterns of behavior, interests, or activities (with at least two manifestations); (C) symptoms present from early childhood, even if not fully evident until social demands exceed capacities; and (D) clinically significant impairment in functioning. The diagnosis encompassed all three DSM-5 severity levels, ranging from Level 1 (“requiring support”) to Level 3 (“requiring very substantial support”). Specifically, 7 individuals were diagnosed with ASD Level 1, 15 with ASD Level 2, and 16 with ASD Level 3. Additionally, for 15 patients, the ASD level was not specified. In this study, we utilized the Autism Diagnostic Observation Schedule, Second Edition (ADOS-2) [15], which is widely recognized instruments for the evaluation and diagnosis of ASD.

### 2.2. Next-Generation Sequencing

DNA extraction and purification were performed from patients’ and parents’ peripheral blood leukocytes, following previously established protocols [16]. The genetic panel was designed based on the first 74 genes associated with ASD, selected from the SFARI Gene database (https://gene.sfari.org/) (accessed on 15 October 2019). Genes were selected based on the SFARI scores of 1, 1S, and 2, prioritizing those with the highest number of reported variants for ASD or neurodevelopmental disorders in the HGMD database. A detailed list of the genes harboring the genetic variants, including their chromosomal locations and corresponding SFARI scores, is provided in Table S1. Next-generation sequencing (NGS) was conducted using the Ion Torrent PGM platform for patients and both their parents. Template preparation, clonal amplification, recovery, enrichment of template-positive Ion Sphere™ Particles, and loading onto Ion 314 semiconductor chips were performed using the Ion Chef™ System. Sequencing was carried out with the Ion S5 Sequencing Kit (Thermo Fisher Scientific, Waltham, MA, USA). Post-run data processing was conducted using Ion Torrent Suite 5.10, Variant Caller 5.10, Coverage Analysis 5.10, and Ion Reporter. The reference genome used was hg19. Variants were filtered according to the following criteria: (i) recessive, de novo, or X-linked inheritance patterns; (ii) minor allele frequency (MAF) < 1%, based on the 1000 Genomes, ESP6500, ExAC, and GnomAD databases. Variant filtering and prioritization were performed using VarAft software version 2.13, and candidate variants were visualized with the Integrated Genomics Viewer (IGV). All candidate variants were validated by Sanger sequencing. The selected variants were classified according to the guidelines established by the American College of Medical Genetics and Genomics (ACMG) [17], using the Varsome platform [18]. Variant classification was performed using a point-based scoring system, where variants were classified as benign if they scored less or equal than −4 points, likely benign for scores between −3 and −1, variants of VUS for scores ranging from 0 to 5, likely pathogenic for scores between 6 and 9, and pathogenic for scores of 10 or more. Individuals were considered positive only if they carried variants classified as likely pathogenic or pathogenic.

### 2.3. Data Analysis

Data were analyzed using R studio software version 3.4.3. Specifically, the packages used were ggplot2 and plotrix. The DOMINO tool (https://domino.iob.ch/) (accessed on 7 July 2025) was utilized to predict the inheritance patterns of genes harboring the identified genetic variants, with scores close to 1 indicating autosomal dominant and scores close to 0 indicating autosomal recessive inheritance. The data of the gene expression of the 9 genes harboring pathogenic or likely pathogenic variants were retrieved from BrainRNAseq database (https://brainrnaseq.org/) (accessed on 30 April 2025) and subsequently elaborated using R studio software version 3.4.3.

## 3. Results

**Figure 1:**
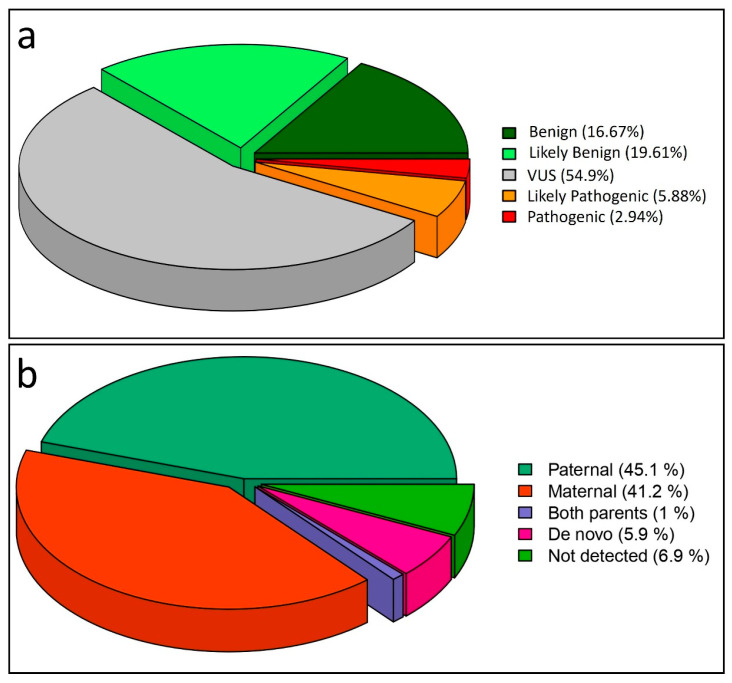
Pie charts illustrating variant classification across the examined cohort, and the differential inheritance. (**a**) Classification of the 102 genetic variants identified across the 53 examined ASD individuals. Dark green indicates benign variants; light green illustrates the likely benign variants; grey depicts the variant of uncertain significance (VUS); orange illustrates the likely pathogenic variants; red depicts the pathogenic variants. (**b**) Pie chart illustrating the inheritance of the 102 variants found in this study.

A total of 102 genetic variants with a minor allele frequency (MAF) below 1% were identified across the 53 patients analyzed, within the 74 genes included in the genetic panel. The male-to-female ratio among individuals who underwent genetic panel testing was 5.66:1. Variants were found in 45 genes across the total number of 74 genes. Table S1 reports all the genetic variants associated with ACMG criteria classification for each subject examined in this study. Additionally, Table S1 reports the comorbidities, when present, for all examined subjects, indicating whether they underwent other genetic tests prior to the targeted gene panel. This table also provides the inheritance patterns predicted by DOMINO for each gene as well as the dbSNP and ClinVar annotations for all the variants. Notably, no variants were detected in seven individuals, based on the variant calling and filtering criteria applied. Among the 102 variants identified, 17 (16.67%) were classified as benign, 20 (19.61%) as likely benign, 56 (54.90%) as variants of uncertain significance (VUS), 6 (5.88%) as likely pathogenic, and 3 (2.94%) as pathogenic (Figure 1).

Among the nine likely pathogenic and pathogenic variants, seven were identified in males, while two were identified in females. Regarding variant inheritance, 46 variants (45.10%) were of paternal origin, while 42 (41.18%) displayed maternal inheritance. Six variants (5.88%) were identified as de novo, and for seven variants (6.86%) located in the genes *ASH1L*, *NLGN3*, *SMARCC2*, *CUX1*, *KMT2C*, *KMT5B*, and *GRIN2B*, inheritance could not be determined due to the unavailability of parental samples. Notably, a single variant in the *MED13* gene was inherited in homozygous form from both parents.

The de novo variants were found in the following genes: *POGZ* (2 variants), *NCOR1*, *CHD2*, *ADNP*, and *GRIN2B*. Among these, two were classified as variants of VUS: *POGZ* (c.1352C>T) and *NCOR1* (c.3409G>A); one was likely pathogenic: *GRIN2B* (c.2141T>A); and three were pathogenic: *POGZ* (c.2323_2324delCT), *CHD2* (c.3323_3324delCT), and *ADNP* (c.14delC) (Table 1).

Among the 53 patients analyzed, nine individuals carrying likely pathogenic or pathogenic variants were classified as “positive” for the genetic test (Table 2). The positive detection rate of the genetic panel adopted on this study was 16.98%.

Conversely, 11 patients were classified as “negative”, due to the absence of any VUS, likely pathogenic, or pathogenic variants. Of these, 7 patients showed no detectable variants according to the applied filtering criteria, while the remaining 4 exhibited only benign or likely benign variants.

Among all the examined patients, 7 were diagnosed with ASD level 1; all were classified as VUS cases, as they did not carry any pathogenic or likely pathogenic variants but had at least one variant of uncertain significance. Of the 15 patients with ASD level 2, 9 were classified as VUS, 2 carried pathogenic or likely pathogenic variants (positive results), 1 had a negative result, and 3 had no available genetic test results. Among the 16 patients with ASD level 3, 9 were categorized as VUS, 3 had positive results, 2 had negative results, and 2 had no genetic test results. Finally, among the 15 patients for whom ASD severity level was not specified, 8 were described as VUS, 4 had positive results, 1 had a negative result, and 2 had no genetic test results.

**Figure 2:**
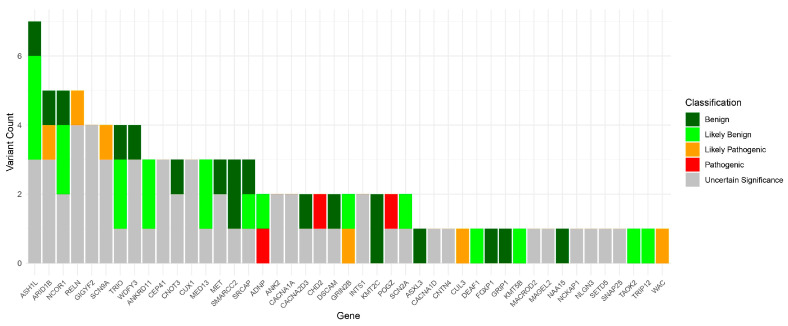
Distribution of the genetic variants identified in this study across the 45 genes of the target genetic panel in which they were found. Dark green was used for benign variants, light green for likely benign variants, orange for likely pathogenic variants, red for pathogenic variants, and gray for variants of uncertain significance (VUS).

In terms of chromosomal distribution, chromosome 7 exhibited the highest number of variants, with a total of 18. Among these, 3 were benign, 14 were classified as VUS, and 1 as likely pathogenic. At the gene level, *ASH1L* harbored the greatest number of variants (*n* = 7), including 1 benign, 3 likely benign, and 3 pathogenic variants. Figure 2 illustrates the distribution of the variants found in this study across the 45 genes in which variants have been found.

## 4. Discussion

In this study, we investigated 53 patients meeting DSM-5 criteria for autism spectrum disorder (ASD) using a customized 74-gene panel based on the SFARI database. Variants were found in 45 genes across the total number of 74 genes. According to the SFARI Gene scoring system, which categorizes genes based on the strength of evidence linking them to autism spectrum disorder (ASD)—with score 1 indicating high-confidence genes, score 2 indicating strong candidates and score S denoting syndromic genes associated with well-established genetic syndromes—the 45 genes in which genetic variants were found in our panel are distributed as follows: 15 genes (*ANK2*, *ASH1L*, *CACNA2D3*, *CUL3*, *DSCAM*, *GIGYF2*, *GRIN2B*, *KMT5B*, *NCKAP1*, *NLGN3*, *RELN*, *SCN2A*, *SRCAP*, *TRIO*, *WDFY3*) are classified as score 1 (high confidence); 11 genes (*CACNA1D*, *CEP41*, *CNTN4*, *CUX1*, *GRIP1*, *MACROD2*, *MET*, *NCOR1*, *SCN9A*, *SNAP25*, *TAOK2*) as score 2 (strong candidates); 18 genes (*ADNP*, *ANKRD11*, *ARID1B*, *ASXL3*, *CACNA1A*, *CHD2*, *CNOT3*, *DEAF1*, *FOXP1*, *KMT2C*, *MAGEL2*, *MED13*, *NAA15*, *POGZ*, *SETD5*, *SMARCC2*, *TRIP12*, *WAC*) as score 1S (syndromic); and *INTS1* as score S (syndromic). The genes *ADNP*, *DEAF1*, *DSCAM*, *GRIN2B*, and *RELN* have been previously reported to be associated with ASD and are expressed in the amygdala [19].

The male-to-female ratio observed in our study was 5.56, which is consistent with previous findings, such as a reported ratio of 4.4 in the Italian population [20]. Our analysis yielded a diagnostic detection rate of about 17%, consistent with the published literature. Notably, these findings align with a previous study of 100 ASD children that reported a 12% diagnostic yield using a comprehensive approach combining a 161-gene panel, chromosomal microarray, and fragile X testing [21]. These findings contrast with the higher diagnostic yield (~25%) achievable through contemporary clinical exome sequencing approaches [22], highlighting the evolving landscape of ASD genetic testing. It is worth mentioning that, for a subset of patients, CGH-array and *FMR1* genetic analyses were performed. As reported in the Supplementary Material, CGH-array identified copy number variations (both deletions and duplications) in specific genomic regions. Nevertheless, although we cannot exclude a potential contribution of these alterations to the patients’ phenotypes, we believe they are unlikely to represent the sole cause of ASD in these individuals. In fact, when performed, CGH-array was always followed by the targeted gene panel analysis described in this study. In addition, *FMR1* genetic analyses, when performed, yielded normal results. To date, whole genome sequencing (WGS) has demonstrated a higher diagnostic yield compared to WES and other genetic tests. As previously documented, WGS achieves a diagnostic yield of approximately 41.1% for neurodevelopmental disorders, including autism [23]. However, a limitation of this approach is that data analysis is more complex, and the overall costs are currently higher than those associated with WES [24].

The custom panel identified 102 ultra-rare variants (MAF < 1% in general populations), all representing private mutations (not recurrent in our cohort). While these variants cannot be classified as direct ASD causes, we posit that they may constitute potential predisposition factors that collectively modulate disease phenotype through oligogenic effects [24,25] or interactions with environmental triggers [26,27]. We included likely benign and benign variants in our analysis because the affected genes are listed in the SFARI Gene database, underscoring their potential relevance despite the current ACMG classifications. Although these variants are currently considered non-pathogenic or of uncertain significance, future evidence may prompt their reclassification. All variants studied are rare in the general population (MAF < 1%). Including them offers a more comprehensive evaluation of the genetic panel’s diagnostic yield.

Overall, the genetic variants identified in this study predominantly localize to well-characterized ASD-associated chromosomal regions, with significant clustering observed on chromosomes 2 (13 variants) and 7 (18 variants). These findings reinforce existing evidence from large-scale genomic studies [28,29] that these loci represent established ASD susceptibility regions, particularly highlighting chromosome 7’s prominent role in neurodevelopmental pathogenesis. Based on various hypotheses, chromosome 7 plays a significant role in autism because it contains many genes involved in the development and functioning of the central nervous system, as well as in language processing [30,31]. Alterations in these genes may contribute to the autistic phenotype. Numerous genetic association studies and linkage analyses have identified significant signals in specific regions of chromosome 7—particularly from 7q11 to 7q31—that are linked to neurodevelopmental disorders such as autism [32,33].

Although NGS has significantly advanced our understanding of ASD by enabling the identification of rare genetic variants associated with the condition, a substantial proportion of these variants identified in clinical settings are still classified as VUS. This reflects the ongoing limitations in variant interpretation and genotype-phenotype correlation. In this study, we report that 54.9% of all identified rare variants in our ASD cohort were classified as VUS. VUS represent a major challenge in genomic medicine, as they create diagnostic ambiguity, complicate genetic counseling, and often contribute to a prolonged “diagnostic odyssey” for families seeking answers [34]. In our cohort, we identified multiple VUS across 32 genes previously implicated in neurodevelopmental processes, synaptic function, and ASD susceptibility. These genes include *WDFY3*, *MET*, *DSCAM*, *POGZ*, *CUX1*, *ANK2*, *MACROD2*, *CACNA1D*, *TRIO*, *NLGN3*, *NCOR1*, *SCN2A*, *ARID1B*, *SNAP25*, *MAGEL2*, *SMARCC2*, *ASH1L*, *RELN*, *SRCAP*, *CEP41*, *CNTN4*, *GIGYF2*, *SETD5*, *SCN9A*, *CACNA2D3*, *NCKAP1*, *MED13*, *ANKRD11*, *CHD2*, *CNOT3*, *CACNA1A*, and *INTS1*. All of these are included in the SFARI Gene database and are expressed in critical brain regions involved in social cognition and behavioral regulation, such as the amygdala and prefrontal cortex, further supporting their potential relevance in ASD pathophysiology [19,35]. Although these variants do not currently meet ACMG criteria for definitive pathogenicity, their presence in genes associated with neuronal migration, synaptic plasticity, chromatin remodeling, and signal transduction highlights their potential contribution to the complex polygenic architecture of ASD. For instance, *CACNA1A* encodes calcium channels essential for synaptic transmission and neuronal excitability [36], while *DSCAM* and WDFY3 are involved in neurodevelopmental pathways critical for neuronal wiring and connectivity [37,38]. Additionally, as annotated in QuickGO database related to the gene ontology (GO), *MET*, *CUX1*, *NLGN3*, *MACROD2*, *CACNA1D*, *TRIO*, *NCOR1*, *SCN2A*, *SNAP25*, and *MAGEL2* participate in diverse pathways related to synaptic function, chromatin modification, and cellular signaling, emphasizing their potential involvement in ASD-related neurodevelopmental processes. While *ASH1L* harbored the highest number of variants overall, only *RELN* contained multiple VUS (*n* = 4)—a notable finding given its established role in ASD pathogenesis. Although currently classified as VUS, these *RELN* variants may be reclassified as likely pathogenic with additional evidence (e.g., functional assays or segregation studies), highlighting how VUS in high-risk ASD genes represent valuable targets for both diagnostic reinterpretation and mechanistic research, despite their indeterminate clinical significance at present [39].

As documented, de novo variants may contribute to ASD pathogenesis [40,41]. This study identified de novo variants in established ASD-associated genes, with pathogenic/likely pathogenic variants supporting ASD’s genetic basis. Notably, variants of VUS in *POGZ* and *NCOR1* (Table 1) warrant attention—while not definitively causative, their presence in known ASD genes and autosomal dominant inheritance suggest potential pathogenicity, consistent with bioinformatic predictions. In fact, both variants received a score of 5 points according to the ACMG variant classification scoring system. As per this system, variants reaching 6 points are classified as likely pathogenic, indicating that these two variants are near the threshold for reclassification.

The nine pathogenic and likely pathogenic variants identified in nine different individuals were located across the genes encoding: Activity-Dependent Neuroprotector Homeobox Protein (ADNP), AT-Rich Interaction Domain 1B (ARID1B), Chromodomain Helicase DNA Binding Protein 2 (CHD2), Cullin 3 (CUL3), Glutamate Ionotropic Receptor NMDA Type Subunit 2B (GRIN2B), Pogo Transposable Element Derived With ZNF Domain (POGZ), Reelin (RELN), Sodium Voltage-Gated Channel Alpha Subunit 9 (SCN9A), and WW Domain Containing Adaptor With Coiled-Coil (WAC). All of them, were previously associated with neurodevelopmental features, as annotated in OMIM database. Table 3 lists the association of these nine genes with neurodevelopmental disorders, indicating the respective MIM phenotype number.

Likely pathogenic and pathogenic variants inherited from parents were identified in several genes, including *ARID1B*, *CUL3*, *RELN*, *SCN9A*, and *WAC*, and will be further analyzed in our study. At our institute, ASD diagnosis is first established in affected children before parental history is considered. Currently, most parents carrying these variants remain undiagnosed, and their unaffected status cannot be definitively confirmed. Although this study focused primarily on affected children, we cannot exclude the presence of subtle parental endophenotypes, which were not assessed given that the study’s scope was limited to ASD diagnosis in children using a targeted gene panel. We cannot rule out the possibility of broader autism phenotypes (BAPs), which encompass a range of sub-diagnostic autistic traits that are more prevalent in families of individuals with ASD than in the general population. BAP is a latent construct with variable definitions and measurement tools, leading to differing prevalence estimates across studies, with higher prevalence reported in fathers than mothers [42,43,44]. We underscore that understanding parental BAP could help clarify the heterogeneity of ASD etiology and inform parent-mediated interventions. Additionally, the genes *ARID1B*, *CUL3*, *RELN*, *SCN9A*, and *WAC* exhibit complex patterns of penetrance and expressivity that complicate clinical interpretation. *ARID1B* typically shows high penetrance but with a broad phenotypic spectrum, ranging from severe disorders such as Coffin–Siris syndrome to milder or subclinical presentations consistent with BAP traits [45]. *RELN* variants demonstrate incomplete penetrance and variable expressivity, where biallelic mutations cause severe lissencephaly, while monoallelic variants are linked to increased ASD risk with subtler features [46,47]. *CUL3* and *SCN9A* also display incomplete penetrance and phenotypic variability [48,49]. Particularly, *CUL3* can be influenced by genetic and environmental modifiers determining potential phenotype-associated episignature [50]. WAC mutations cause a well-characterized syndrome with generally high penetrance, yet notable phenotypic variability, often including ASD-related traits [51]. This complexity highlights that pathogenic or likely pathogenic variants in these genes do not consistently produce a uniform clinical phenotype, underscoring the need for integrated evaluations that consider incomplete penetrance and variable expressivity. Familial segregation studies and functional analyses are essential to improve diagnostic precision and advance our understanding of the genetic contribution of these genes to ASD pathogenesis. Additionally, we cannot rule out the involvement of genetic modulators that may influence neurodevelopmental processes contributing to the observed phenotypes in the patients [52].

**Figure 3:**
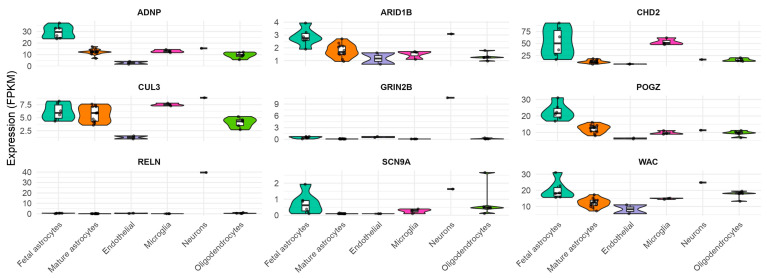
Violin plots displaying FPKM (fragments per kilobase million) expression levels for the nine genes harboring pathogenic or likely pathogenic variants identified in this study. Expression profiles across neural cell types were obtained from the BrainRNAseq database (https://brainrnaseq.org/) (accessed on 10 June 2025), revealing cell-type-specific expression patterns for each ASD-associated gene.

Despite the fact that several of these genes are not directly causative for ASD, they involve cerebral pathways closely associated with ASD. In fact, according to the QuickGO annotations, these genes converge on three key neurodevelopmental pathways: (1) chromatin remodeling (GO:0006338) (*ARID1B*, *CHD2*, *POGZ*, *WAC*), which regulates gene expression during neural differentiation; (2) synaptic signaling (GO:0050805; GO:0051965) (*GRIN2B*, *SCN9A*, *ADNP*), pivotal for neurotransmission and plasticity; and (3) protein homeostasis (GO:0033484) (*ADNP*) and neuronal migration (GO:0097475; GO:1904936) (*RELN*). Notably, 78% (7/9) participate in transcriptional regulation through either direct DNA binding (*ARID1B*, *CHD2*) or epigenetic modulation (POGZ, WAC), highlighting chromatin dysregulation as a central mechanism in these cases. The remaining genes primarily affect synaptic function (*GRIN2B*, *SCN9A*) or structural brain development (*RELN*). Supporting their ASD relevance, *CHD2*, *GRIN2B*, and *CUL3* show high ASD association scores higher than 500 in the MalaCards database (https://www.malacards.org/) (accessed on 10 June 2025). BrainRNAseq (https://brainrnaseq.org/) (accessed on 10 June 2025) database revealed that all nine candidate genes (*ADNP*, *ARID1B*, *CHD2*, *CUL3*, *GRIN2B*, *POGZ*, *RELN*, *SCN9A*, and *WAC*) demonstrated high expression patterns across several brain cell types (Figure 3).

Their broad yet cell-type-specific expression profiles support two key conclusions: (1) these genes participate in fundamental neurodevelopmental processes, and (2) their dysregulation may contribute to ASD pathogenesis through distinct cellular mechanisms.

Due to the unclear genetic mechanisms involved in ASD pathogenesis, we emphasize that, nowadays, a genetic panel is not the most suitable method for clinical testing for ASD. In fact, a potential limitation of this study is that our NGS panel analysis was restricted to 74 known autism-associated genes, which may have prevented the identification of novel ASD-related genes. The patients who resulted negative to the genetic test performed in this study might harbor variants in established ASD genes not included in our panel, or novel genes not yet associated with autism. We emphasize that this study was conducted as part of a research project started in 2018, during which targeted gene panels represented a standard and clinically validated approach for ASD genetic testing, prior to the widespread adoption of WES in routine diagnostics. We are aware that the targeted genetic panel evaluated in this study has now largely been surpassed by WES in the genetic evaluation of ASD. Nevertheless, we consider it still useful for research purposes, as it enables the identification of novel genetic variants and de novo mutations associated with patient phenotypes. As demonstrated in this study, the use of a targeted panel allowed us to identify multiple rare and de novo variants in genes relevant to ASD, contributing to the expansion of the genetic landscape associated with the disorder. WES addresses this diagnostic limitation by interrogating all protein-coding regions (and consequently demonstrates higher ASD mutation detection rates), current genomic approaches remain constrained by several factors: the clinical heterogeneity of ASD, inconsistencies in the ASD genetics literature, and potential pathogenic contributions from non-coding genomic regions and epigenetic mechanisms. These limitations underscore that both WES and targeted genetic panel analysis cannot yet deliver definitive autism diagnoses, emphasizing the need for more comprehensive genomic analyses in ASD research. A further limitation of this study is the inability of our targeted sequencing approach to detect multi-nucleotide expansions or repeat sequences, which represent known but mechanistically distinct genetic contributors to ASD pathogenesis [53,54]. This study has expanded the ClinVar database through submissions of the de novo novel variants, enhancing the understanding of ASD’s genetic architecture and providing valuable data for future diagnostic interpretation.

## 5. Conclusions

This study evaluates the diagnostic performance of a customized 74-gene panel in 53 unrelated ASD patients, identifying 102 variants across 45 genes. The gene harboring the highest number of genetic variants was *ASH1L*. The panel achieved a 16.98% diagnostic yield, with nine individuals carrying pathogenic/likely pathogenic variants in *ADNP*, *ARID1B*, *CHD2*, *CUL3*, *GRIN2B*, *POGZ*, *RELN*, *SCN9A*, and *WAC*. Notably, all the de novo variants were submitted to ClinVar, significantly expanding the annotated mutational spectrum of ASD-associated genes. We underscore that special attention should be dedicated to the VUS identified in this study, which accounted for 54.9% of all rare variants detected in our ASD cohort. To date, VUS represent a major challenge in genomic medicine, as they create diagnostic ambiguity and complicate genetic counseling. We hope that these variants can be re-evaluated in the future to expand our understanding of the genetic contributions to ASD.

## Figures and Tables

**Table 1:** List of the de novo variants identified in this study. Table shows the specific patient’s code, gene name, chromosome localization, the nomenclature of the mutation, the ACMG variant classification and the inheritance. All these genes are associated with an autosomal dominant inheritance pattern, with the exception of NCOR1, for which the mode of inheritance could not be determined.

Patient	Gene	Coding	Protein	ACMG	ClinVar
P4	POGZ	c.2323_2324delCT	p.Leu775ValfsTer4	P (PVS1 ^1^, PM2 ^2^, PS2 ^3^)	SCV006076861
P5	POGZ	c.1352C>T	p.Pro451Leu	VUS (BP4 ^4^, PM2, PS2, PP4 ^5^)	SCV006076862
P11	NCOR1	c.3409G>A	p.Gly1137Arg	VUS (BP4, PM2, PS2)	SCV006076865
P17	CHD2	c.3323_3324delCT	p.Ser1108Ter	P (PVS1, PM2, PS2, PP5 ^6^)	SCV001592231
P18	ADNP	c.14delC	p.Pro5fs	P (PVS1, PP5, PS2, PM2)	SCV003803113
P50	GRIN2B	c.2141T>A	p.Val714Glu	LP (PP3, PM1 ^7^, PM2, PS2)	SCV006076866

^1^ PVS1: Loss of function (LOF); ^2^ PM2: Absent from controls; ^3^ PS2: De novo variant; ^4^ BP4: Computational evidence; ^5^ PP4: Phenotype match; ^6^ PP5: reputable source; ^7^ PM1: Mutation hotspot.

**Table 2:** List of the five parental inherited pathogenic (P) and likely pathogenic (LP) variants identified in five diagnosed individuals. All these variants exhibited an autosomal dominant inheritance pattern. Notably, the *RELN* and *SCN9A* genes are also associated with autosomal recessive inheritance.

Patient	Gene	Coding	Protein	Inherithance	ACMG
P29	*ARID1B*	c.4692_4696delTGGCGinsGGGC	p.Gly1566AlafsTer11	Maternal	LP (PVS1 ^1^, PM2 ^2^)
P23	*CUL3*	c.957delC	p.Tyr320IlefsTer2	Maternal	LP (PVS1, PM2)
P47	*RELN*	c.754A>T	p.Ile252Phe	Maternal	LP (PM2, PP2 ^3^, PP4 ^4^, PP5 ^5^)
P33	*SCN9A*	c.2159T>A	p.Ile720Lys	Maternal	LP (BP4 ^6^, PP4, PM2, PM5 ^7^)
P46	*WAC*	c.42-9_42-6delGTTT	p.?	Paternal	LP (PM2, PP3, PP4)

^1^ PVS1: Loss of function (LOF); ^2^ PM2: Absent from controls; ^3^ PP2: Rare benign missense; ^4^ PP4: Phenotype match; ^5^ PP5: reputable source; ^6^ BP4: Computational evidence; ^7^ PM5: Other aa change in same codon.

**Table 3:** List of the nine genes harboring likely pathogenic and pathogenic variants, with the OMIM associated phenotype, as well as the MIM phenotype number and inheritance (autosomal dominant: AD; autosomal recessive: AR).

Gene	Phenotype	MIM	Inheritance
*ADNP*	Helsmoortel-van der Aa syndrome	615873	AD ^1^
*ARID1B*	Coffin–Siris syndrome 1	135900	AD
*CHD2*	Developmental and epileptic encephalopathy 94	615369	AD
*CUL3*	Neurodevelopmental disorder with or without autism or seizures	619239	AD
	Pseudohypoaldosteronism, type IIE	614496	AD
*GRIN2B*	Developmental and epileptic encephalopathy 27	616139	AD
	Intellectual developmental disorder, autosomal dominant 6	613970	AD
*POGZ*	White–Sutton syndrome	616364	AD
*RELN*	{Epilepsy, familial temporal lobe, 7}	616436	AD
	Lissencephaly 2 (Norman-Roberts type)	257320	AR ^2^
*SCN9A*	Erythermalgia, primary	133020	AD
	Insensitivity to pain, congenital	243000	AR
	Neuropathy, hereditary sensory and autonomic, type IID	243000	AR
	Paroxysmal extreme pain disorder	167400	AD
	Small fiber neuropathy	133020	AD
*WAC*	Desanto-Shinawi syndrome	616708	AD

^1^ AD: autosomal dominant inheritance; ^2^ AR: autosomal recessive inheritance.

## Data Availability

The data presented in this study are available upon request from the corresponding author. The data are not publicly available in order to protect the privacy of the patient and the family.

## Supplementary Materials

The following supporting information can be downloaded at https://www.mdpi.com/article/10.3390/medicina61071273/s1, Table S1: List of 102 genetic variants identified in this study, for all 53 individuals examined.

## Abbreviations

The following abbreviations are used in this manuscript:

ACMG	American College of Medical Genetics and Genomics
ASD	Autism spectrum disorder
IGV	Integrated genomics viewer
MAF	Minor allele frequency
NGS	Next-generation sequencing
SFARI	Simons Foundation Autism Research Initiative
VUS	Variant of uncertain significance
WES	Whole exome sequencing
